# Natural History and Predictors for Hemorrhage in Supratentorial Brain Arteriovenous Malformations

**DOI:** 10.3390/jcm13133760

**Published:** 2024-06-27

**Authors:** Ioana Miron, Viorel M. Prună, Dan M. Visarion, George E. D. Petrescu, Radu M. Gorgan

**Affiliations:** 1 Department of Neurosurgery, “Carol Davila” University of Medicine and Pharmacy, 020021 Bucharest, Romania; mironioana@gmail.com (I.M.); dan.visarion@drd.umfcd.ro (D.M.V.); george.petrescu@umfcd.ro (G.E.D.P.); radu.gorgan@umfcd.ro (R.M.G.); 2 Department of Neurosurgery, “Bagdasar-Arseni” Clinical Emergency Hospital, 041915 Bucharest, Romania

**Keywords:** brain arterio-venous malformations, supratentorial AVMs, hemorrhagic risk, predictors of hemorrhage, risk factors for epileptic seizures, unruptured AVMs, venous drainage, small AVMs, pediatric AVMs, birth-to-diagnosis method

## Abstract

**Background/Objectives:** Approximately half of the patients harboring supratentorial brain arterio-venous malformations (stAVMs) present with hemorrhage, and another considerable proportion suffer from epileptic seizures. An important milestone in the management of this vascular pathology is acknowledging their natural history, especially across long periods of time. The aim of this study was to assess the predictive factors for hemorrhage and for epileptic seizures as presenting symptoms in stAVMs. **Methods:** We retrospectively analyzed patients with stAVMs admitted to our institution between 2012 and 2022 and evaluated predictive factors for hemorrhage and the risk factors associated with epileptic seizures. **Results:** The cohort included 169 patients, 78 of them (46.2%) presenting with intracerebral hemorrhage (ICH). Seventy-seven (45.5%) patients suffered from epileptic seizures. The annual hemorrhagic rate was 1.28%/year. Unruptured lesions (*p* = 0.001, OR 3.1, 95% CI 1.6–6.2), superficial venous drainage (*p* = 0.007, OR 2.7, 95% CI 1.3–5.7) and large nidus size (*p* = 0.025, OR 4, 95% CI 1.2–13.5) were independently associated with seizures. Among unruptured lesions, superficial venous drainage (OR 2.6, *p* = 0.036, 95% CI 1.06–6.3) and frontal/temporal/parietal location (OR 2.7, *p* = 0.040, 95 CI% 1.04–6.9) significantly increased the risk of seizures as a presenting symptom in multivariate analysis. Patients younger than 18 (*p* = 0.003, OR 4.5, 95% CI 1.6–12.2), those with AVMs < 3 cm (*p* = 0.03, OR 2, 95% CI 1.07–3.9) or those with deep located AVMs (*p* = 0.035, OR 2.3, 95% CI 1.06–5.1) presented statistically more often with ICH in multivariate regression. Small size (HR 1.8, 95% CI 1.09–3, *p* = 0.022) and exclusively deep venous drainage (HR 2.2, 95% CI 1.2–4, *p* = 0.009) were independent predictors for ICH, in time-dependent birth-to-diagnosis analysis. After shifting the birth-to-diagnosis curve by 10 years, unique arterial feeder demonstrated a positive correlation with ICH presentation as well. **Conclusions:** Small AVMs, those with exclusively deep venous drainage, unique arterial feeder or deep location may pose higher hemorrhagic risks for the patient, and therapeutic strategies should be tailored accordingly. When managing unruptured brain AVMs, it is important to consider the risk of developing seizures, in addition to the lifelong risk of hemorrhage, in determining the optimal treatment approach for each patient.

## 1. Introduction

Brain arterio-venous malformations (AVMs) are vascular lesions comprised of abnormal tortuous arteries and veins without intervening capillaries, forming a vascular nidus. The flow disturbances generated by high-pressure arterial blood arriving directly into the draining veins are the cause of their most frequent presenting symptom, intracerebral hemorrhage (ICH). Hemorrhage represents half of all presenting symptoms in some series [1,2], and although infratentorial AVMs are known to have much higher rupture rates, supratentorial AVMs (stAVMs) still have more than 50% incidence of ICH presentation in some series [3].

Seizures are the second most common symptom in stAVMs’ natural history, occurring in 30% of cases in the series of Englot et al., with 18% of patients who presented for seizures progressing to medically refractory epilepsy [4]. Given the high impact of living with epilepsy on the patient’s quality of life, it is important to assess predicting factors for developing seizures in brain AVMs and the best therapeutic approach to achieving seizure freedom.

The present study aims to evaluate the natural history of stAVMs. We decided to exclude infratentorial lesions because they represented a particular subgroup with different hemorrhagic rates and no epileptogenic symptoms.

Gaining insight into the natural history of these challenging vascular lesions, especially through extensive follow-up studies, can help clinicians improve the therapeutic algorithm. AVMs are rare lesions, and data regarding long term-natural history of untreated brain AVMs is scarce. For this reason, we decided to use the birth-to-diagnosis method for assessing hemorrhage rate and the predicting factors for hemorrhage in stAVMs. According to Kim et al., this method offers similar results to the conventional diagnosis-to-ICH method [5].

## 2. Materials and Methods

### 2.1. Patient Selection

**Figure 1:**
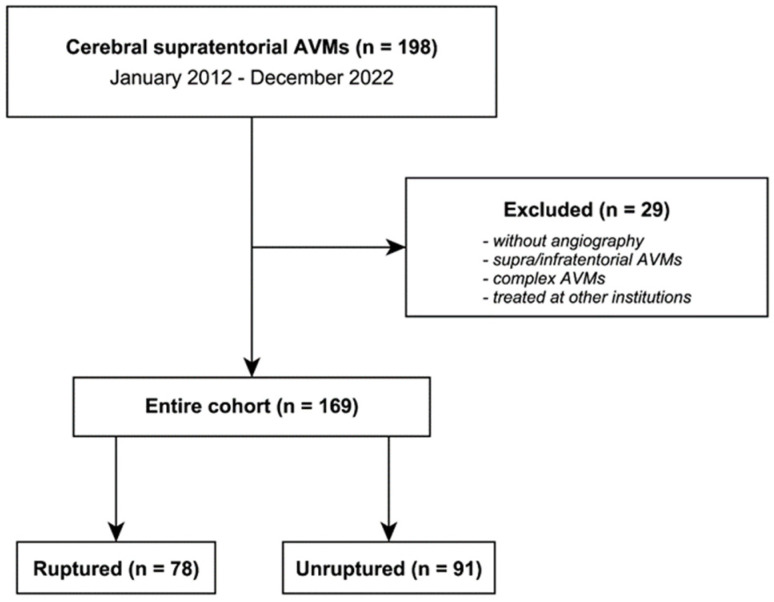
Flowchart illustrating the design of the study.

Between January 2012 and December 2022, 198 patients with stAVMs were admitted to the “Bagdasar-Arseni” Emergency Clinical Hospital. After excluding patients without angiographic investigations, supra/infratentorial AVMs, complex AVMs (multiple AVMs, AVMs in association with AVFs) and patients treated before at other institutions, the final cohort of the observational single-center retrospective study had 169 patients harboring stAVMs. Figure 1 depicts the flowchart of the study.

### 2.2. Baseline Data

Patients’ demographic data, as well as their clinical and imaging characteristics, were retrospectively assessed.

We recorded the presenting neurologic condition and symptoms, the hemorrhagic status at diagnosis and during follow-up, and the occurrence of intraventricular hemorrhage (IVH). We gathered morphologic details regarding AVMs: size (divided in three categories: nidus < 3 cm, between 3–6 cm, and nidus > 6 cm), location, eloquent area involvement, venous drainage pattern, the number of arterial feeders, the number of drainage veins, and associated aneurysms. Deep location was defined as any AVM that was not cortical or immediately underneath the cortical surface, including those in the deep white matter, internal capsule, thalamus, deep cerebral nuclei, deep cerebellar nuclei, brainstem, ventricular system, cerebral peduncles, etc. Spetzler–Martin (SM), Lawton–Young (LY), Spetzler–Ponce (SP) and Supplementary SM grades were noted for every lesion. The diagnosis of the AVM was achieved using digital subtraction angiography (DSA), CT angiography (CTA) and MR angiography (MRA).

The hemorrhagic rate was calculated using the following formula: (number of patients with ICH event)/(total number of patient years of follow-up) × 100 [5].

### 2.3. Statistical Analysis

IBM SPSS Statistics for Windows, version 29 (IBM Crp., Armonk, NY, USA) and GraphPad Prism version 10.2.2 for Windows (GraphPad Software, San Diego, CA, USA) were used for statistical analysis and graphic representation. Pearson chi-square test and, when appropriate, Fisher exact test were used in univariate analysis to compare categorical variables. Statistical significance was considered at *p* < 0.05. Multivariate logistic regression analysis was used to assess the factors independently associated with hemorrhagic presentation, introducing only variables that were significant in univariate analysis. The log-rank test, as part of the Kaplan–Meier analysis, was used to evaluate the significance of different factors in relation to the ICH presentation, using the birth-to-diagnosis method. Cox proportional hazards regression was employed to evaluate independent risk factors for hemorrhagic presentation.

## 3. Results

The demographic and clinic baseline characteristics of the 169 patients included in the study are summarized in Table 1.

Based on angiographic studies (DSA 91.1%, CTA 1.8%, MRA 7.2%), we documented different morphological characteristics and their distribution in the entire cohort. The median SM grade was 3 (1–5). The radiologic characteristics are summarized in Table 2. Of the 23 cases (13.6%) harboring aneurysms associated with an AVM, 12 cases presented intranidal aneurysms, 10 had flow-related aneurysms, and one patient had many types of aneurysms.

### 3.1. Treatment

Of the 169 cases, 69 (40.8%) were treated with microsurgical resection, 19 (11.2%) with endovascular treatment, 33 with SRS (19.5%), and 48 cases (28.5%) were managed conservatively. Surgical patients were operated on shortly after diagnosis or in the following 1–3 months. Six patients of the SRS group were treated during the follow-up period, months after diagnosis (between 3 months and one year). One patient diagnosed with an unruptured AVM refused treatment. After nine years the case received endovascular treatment, after a first hemorrhagic event. One patient who presented for a ruptured AVM had another hemorrhagic episode 7 months after diagnosis, a few days before the endovascular treatment. She was treated with SRS. Seventeen patients underwent multimodal treatment (combinations between surgical, endovascular and SRS treatment), performed in the follow-up period, if residual nidus was detected after the first therapeutic option and an additional treatment option was feasible.

### 3.2. Follow-Up

The median follow-up time was 14.5 months (range 1–137). There were five cases who suffered hemorrhagic events during follow-up. One case was a SM II unruptured AVM, who refused treatment and presented with ICH 9 years later (Table 1). The other four AVMs were ruptured AVMs: one was first treated with microsurgical resection, presenting a small residual nidus postoperatively that hemorrhaged again nine years later and was treated with SRS; one case had a second hemorrhagic event four years following endovascular treatment and another case bled 7 months after the initial diagnosis, before the endovascular procedure; one conservatively managed ruptured SM III AVM presented with hemorrhage during follow-up two times, four and five years after the diagnosis.

## 4. Hemorrhagic Presentation

### 4.1. Hemorrhagic Rate Analysis

The birth-to-diagnosis period included 6.054 patient years, and the study had 78 AVMs diagnosed with ICH, so the hemorrhagic rate was 1.28/year for previously unruptured AVMs.

### 4.2. Factors Associated with Hemorrhagic Presentation

Small AVMs (diameter < 3 cm) ruptured more often than the others, with 51 out of 91 (56%) small AVMs being diagnosed with hemorrhage (*p* = 0.005).

Age showed a positive correlation with the hemorrhagic status, with the pediatric group being more frequently diagnosed with rupture (*p* < 0.001). A total of 21 out of 27 (77.7%) pediatric patients presented with ruptured AVMs.

A mild significant correlation was observed between eloquent locations and hemorrhagic presentation (*p* = 0.040). There were 112 AVMs in eloquent locations, and 58 (51.8%) of them were diagnosed with hemorrhage, while only 20 (35%) of the 57 non-eloquent AVMs presented in the same manner.

AVMs were also divided into cortical and deep lesions, and out of the 38 deeply situated stAVMs, 24 (63.1%) were diagnosed ruptured (*p* = 0.017). By comparison, there were 131 cortical AVMs, and hemorrhagic presentation was significantly lower in this group, counting 54 (41.2%) patients.

The description of the venous drainage pattern included the location (superficial/deep) and number of drainage veins (unique versus multiple). Exclusively deep venous drainage (*p* = 0.050) and unique venous drainage (*p* = 0.049) were more frequently associated with hemorrhage, but the statistical correlation was borderline significant.

**Figure 2:**
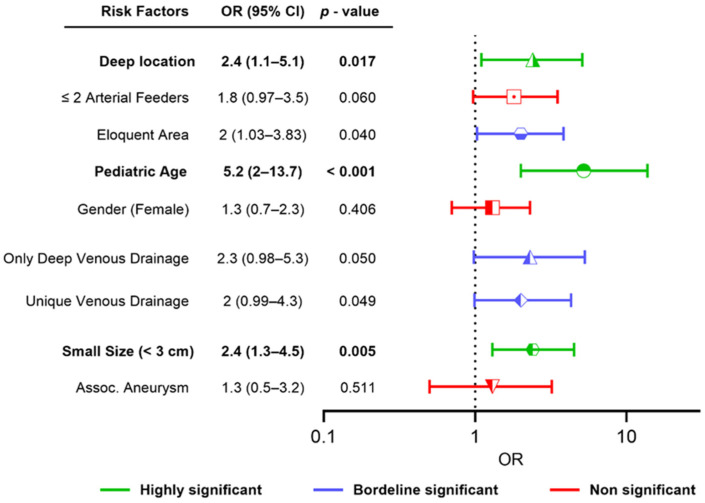
Univariate analysis of predictive factors for hemorrhagic presentations in stAVMs. Deep location, pediatric age and small nidus size < 3 cm were the most significant factors.

Variables like gender, presence of an arterial aneurysm, and the number of arterial feeders (≤2 and >2) have been tested in relation to the rupture status but did not reveal a statistic correlation (OR and 95% CI of the tested variables can be seen in Figure 2).

### 4.3. Multivariate Analysis of Predictors of Hemorrhage

Due to the relatively small sample size, we chose to introduce, in logistic regression, only the variables that proved statistical correlation in univariate analysis, *p* < 0.050. Unique venous drainage was excluded due to borderline results (*p* = 0.049, unreliable 95% CI that do not exclude 1) and because it was highly correlated with size < 3 cm (*p* = 0.003, OR 3, 95% CI 1.4–6.3). Moreover, an exact description was available only in 148 cases.

All the three variables that were introduced in the logistic regression proved to be independently associated with hemorrhagic presentation: size under 3 cm (*p* = 0.03, OR 2, 95% CI 1.07–3.9), pediatric age (*p* = 0.003, OR 4.5, 95% CI 1.6–12.2) and deep location (*p* = 0.035, OR 2.3, 95% CI 1.06–5.1).

## 5. Kaplan–Meier Birth-to-Diagnosis Analysis

We investigated the influence of various factors on the likelihood of a stAVM presenting with hemorrhage. In our analysis, the patient’s age served as the temporal variable, with hemorrhagic presentation being the event of interest. Thus, our aim was to assess the probability of an AVM remaining free from intracerebral hemorrhage (ICH) throughout the patient’s lifespan until diagnosis, considering a range of contributing factors (Table 3).

Small AVMs (<3 cm) were associated with earlier hemorrhagic presentation compared to lesions with medium-to-large size nidus (*p* = 0.017). Exclusively deep venous drainage demonstrated strong correlation with younger ICH presentation (*p* < 0.001). Unique venous drainage (*p* = 0.058), deep located nidus (*p* = 0.052), and unique arterial feeder (*p* = 0.057) revealed some differences, but did not reach statistical significance. Gender (*p* = 0.103), eloquence (*p* = 0.476) and the presence of aneurysms (*p* = 0.543) did not influence the probability of AVM’s hemorrhagic presentation in the Kaplan–Meier analysis.

## 6. Cox PH Regression Analysis

Cox regression proportional hazards analysis was performed to identify independent predictors of early hemorrhagic presentation, with the patient’s age as the time variable and hemorrhage as the event of interest. Size < 3 cm, deep located nidus and exclusively deep venous drainage were introduced in the analysis. Exclusively deep venous drainage demonstrated the greatest impact, resulting in a 2.2 times higher risk of hemorrhage as the initial presentation compared to the superficial or mixed venous drainage pattern (HR 2.2, 95% CI 1.2–4, *p* = 0.009). Patients with small AVMs had a significantly higher risk of presenting with hemorrhage compared to those that harbor larger AVMs (HR 1.8, 95% CI 1.09–3, *p* = 0.022). Deep location did not reach statistical significance when adjusting for the other covariates (HR 1.3, 95% CI 0.73–2.31, *p* = 0.361).

### Shifting the Birth-to-Diagnosis Curve

According to Kim et al. [5], shifting the birth-to-diagnosis timeline by 10 years generates survival curves comparable to those from the diagnosis-to-ICH method. We applied this technique, and the new cohort had 160 patients.

**Figure 3:**
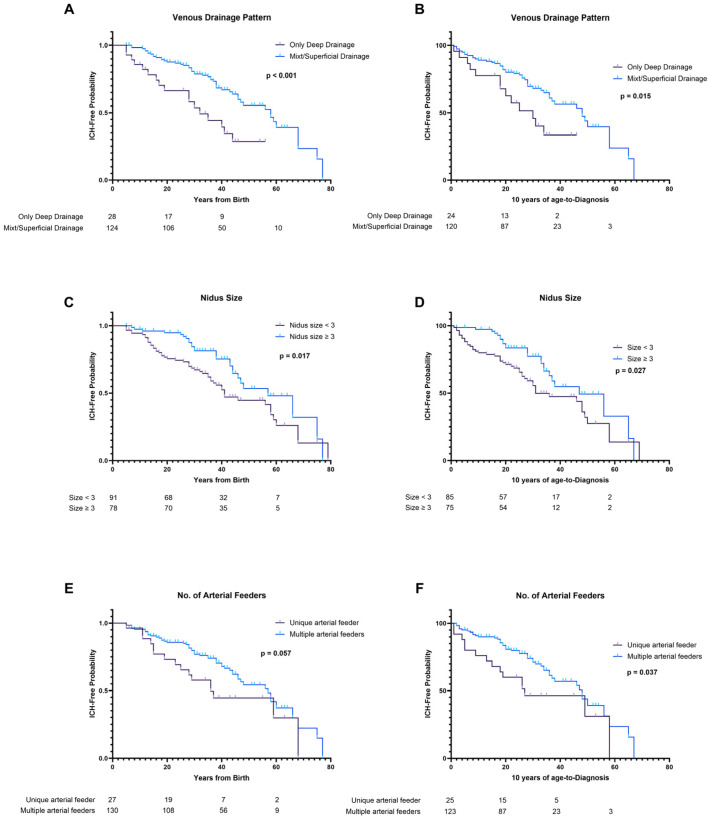
Kaplan–Meier analysis comparing birth-to-diagnosis (**A**,**C**,**E**) survival curves and 10 years of age-to-diagnosis (shifted curves **B**,**D**,**F**) survival curves according to several factors. (**A**,**B**) stAVMs with exclusively deep venous drainage demonstrated reduced ICH-free intervals. (**C**,**D**) Small AVMs were significantly associated with earlier hemorrhagic presentation. (**E**,**F**) AVMs with unique arterial feeder demonstrated reduced ICH-free intervals as well.

Nidus < 3 cm (*p* = 0.027), exclusively deep venous drainage (*p* = 0.015) and unique arterial feeder (*p* = 0.037) proved to significantly influence the hemorrhagic risk in Kaplan–Meier analysis (Table 4). The Kaplan–Meier curves of both methods are demonstrated in Figure 3.

## 7. Epileptic Seizures at Presentation

Seventy-seven patients presented with epileptic seizures. Seizures proved to be statistically correlated in univariate analysis with unruptured AVMs (*p* < 0.001, OR 3.1, 95% CI 1.6–5.9), with size > 6 cm (*p* = 0.029, OR 3.2, 95% CI 1.07–9.5) and with superficial venous drainage (*p* = 0.008, OR 2.3, 95% CI 1.2–4.3). Of the 77 patients presenting with these symptoms, 65 (84.4%) had a superficial location, but univariate analysis demonstrated borderline statistical significance (*p* = 0.049, OR 2.1, 95% CI 0.99–4.5).

**Figure 4:**
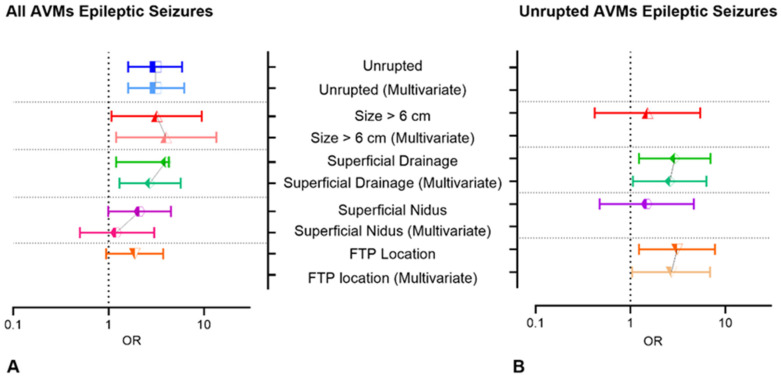
The lines depict the OR variations of different characteristics in univariate and multivariate analysis of risk factors associated with seizures within the entire cohort (**A**), and in the unruptured AVMs subgroup (**B**).

We performed multiple logistic regression, introducing all four covariates, and three of them proved to be independently associated with seizures: unruptured presentation was the most significant, (*p* = 0.001, OR 3.1, 95% CI 1.6–6.2), followed by superficial venous drainage (*p* = 0.007, OR 2.7, 95% CI 1.3–5.7) and large AVMs (>6 cm) (*p* = 0.025, OR 4, 95% CI 1.2–13.5). The OR of univariate and multivariate analyses are depicted in Figure 4A.

### Epileptic Seizures in Unruptured stAVMs

Furthermore, we tested the same risk factors in the unruptured group (Figure 4B), and superficial venous drainage maintained its association with seizures. There were 91 patients diagnosed with unruptured stAVMs, and 53 of them presented with seizures. Thirty-two out of 53 stAVMs (60.37%) had superficial venous drainage, while only 13 (34.21%) of the seizure-free group had the same venous drainage pattern (*p* = 0.014, OR 2.9, 95% CI 1.2–6.9). A nidus larger than 6 cm did not prove statistical correlation (*p* = 0.755), probably due to the small number of cases (12 cases with nidus > 6 cm, and 8 of them presented with seizures). Regarding location, frontal, temporal and parietal locations summed up 63 cases, and 42 of them (79.2%) had epileptic manifestations (*p* = 0.015, OR 3, 95% CI 1.2–7.7). Other locations (occipital, parieto-occipital, temporo-occipital, hemispheric, deep) summed up 28 patients, and only 11 of them presented with seizures.

In multivariate analysis, stAVMs located in frontal/parietal/temporal lobes had a 2.7 higher chance of presenting with seizures (*p* = 0.040, 95 CI% 1.04–6.9), while superficial venous drainage increased the risk of epilepsy by 2.6, in comparison to any deep venous drainage (*p* = 0.036, 95% CI 1.06–6.3).

## 8. Discussion

A comprehensive assessment of brain AVMs’ natural history is an important milestone in establishing the best therapeutic approach for every patient. Ruptured AVMs clearly require treatment to prevent a future hemorrhagic event. These patients often present with an altered neurologic condition due to the brain injury caused by the hematoma. Patients with unruptured AVMs most often present in good clinical condition. Since the ARUBA trial concluded that medical management alone is superior to interventional therapy for the prevention of death and stroke, there has been considerable debate regarding the best treatment of these patients, especially after publishing the final follow-up [6,7].

### 8.1. Hemorrhagic Presentation of Brain AVMs

Given that a hemorrhagic event might significantly alter the patient’s neurologic condition and prognosis [8], we consider it important to evaluate risk factors for hemorrhagic presentation and to adjust therapeutic strategies in unruptured brain AVMs accordingly.

Brain AVMs exhibit different hemorrhagic patterns depending on many factors [9,10,11]. In our cohort, pediatric AVMs presented much more often with rupture, as well as small lesions (<3 cm) and deep AVMs. While pediatric patients tend to present more often with ruptured AVMs [12,13,14,15], a more aggressive character of AVMs in childhood is debatable [11] A possible explanation would be that incidental or mildly symptomatic AVMs are rarely diagnosed in children [12]. In our cohort, 81.3% of pediatric patients presented with ruptured AVMs, similar to the series by Steinberg et al. [15].

Regarding AVM size, many studies stated a positive association between small size (<3 cm) and higher rupture rates [16,17,18]; Spetzler et al. explained it by higher feeding artery pressures in small AVMs [16]. Nevertheless, large data base prospective studies [10] or meta-analysis [19] did not confirm small size as a risk factor for subsequent hemorrhage. Hernesniemi et al. very accurately describe this discrepancy in their long-term follow-up study, stating that small AVMs may be diagnosed more frequently with hemorrhage because larger AVMs become symptomatic sooner, and are diagnosed before rupture [2]. The study outlines the difference between hemorrhagic presentation and risk factors for hemorrhage [2], in a similar way to how Fullerton et al. elaborated on pediatric ruptured AVMs [12]. Nowadays, the increasing availability of MRI scans has led to a rise in the incidental detection of arteriovenous malformations (AVMs). Lu et al. compared the incidental and symptomatic unruptured AVMs in children and found that patients with incidentally discovered lesions had smaller lesions and were younger, compared to those who presented for headaches, or seizures [20], confirming once again the theory that small AVMs may be diagnosed more often with hemorrhage because they do not present with mass-effect related symptoms.

Small or deep AVMs were diagnosed more often with hemorrhage in our cohort, but due to various definitions of deep location across studies, it is difficult to define this anatomic finding as a predictor for hemorrhage [21]. Therefore, we decided to evaluate how several factors might influence a hemorrhagic presentation in time-dependent analysis, for a better understanding of these findings.

### 8.2. Hemorrhagic Rate and Risk Factors for Early Hemorrhagic Presentation

Kim et al. compared the two methods for estimating annual ICH rates in brain AVMs: the birth-to-diagnosis and the diagnosis-to-ICH timelines, and stated that both methods offer similar results [5]. The birth-to-diagnosis method has been used by other authors too [22,23], and it implies calculating the patient years of follow-up, dating from birth. The annual rupture rate for previously unruptured stAVMs using this method was 1.28%. Regarding brain AVMs in general, the “Multicentric AVM Research Study” (MARS) found that the annual rate of hemorrhage for unruptured AVMs was 1.3% [10]. Kim et al. reported an annual rate of 1.27 per 100 patient years using the birth-to-diagnosis method and 1.17% using the diagnosis-to-ICH method [5]. In the meta-analysis of Gross et al. the annual rate was 2.2% [24], while Hernesniemi et al. found a rate of 1.6% [2]. The hemorrhagic rate for stAVMs in our cohort was similar to that reported for AVMs in general. This finding might point out that although the hemorrhagic risk is a major concern in infratentorial brain AVMs [25,26], it should not be neglected in supratentorial AVMs either.

Using the birth-to-diagnosis method, we assessed what factors might contribute to a hemorrhagic presentation, and size < 3 cm and exclusively deep venous drainage significantly influenced the possibility of early hemorrhagic presentation in brain AVMs, both in Kaplan–Meier and in Cox PH regression. The median ICH-free time for patients with exclusively deep venous drainage AVMs was 32 years, compared to 58 years for patients with superficial venous drainage AVMs, exposing the patient to a 2.2 HR of hemorrhagic presentation at diagnosis. Although the association between ruptured AVMs and exclusively deep venous drainage was borderline in univariate analysis (*p* = 0.050), the survival analysis revealed that exclusively deep venous drainage is an independent risk factor for early hemorrhagic presentation in the natural course of brain AVMs. One possible explanation for this higher hemorrhagic risk lies in the anatomical particularities of the deep draining veins, leading to a high impedance. Kellner et al. revealed that this type of draining vein often presents stenosis and has a smaller diameter compared to superficial veins [27].

According to Kim et. al., the birth-to-diagnosis and the diagnosis-to-ICH methods are most similar when shifting the birth-to-diagnosis curve by 10 years, possibly due to a biologic change around this age, that might influence the hemorrhagic rate [5]. We adjusted the timeline accordingly, and nine patients were excluded from the analysis. Despite these adjustments, small size and exclusively deep venous drainage maintained their significance in the Kaplan–Meier analysis. Moreover, the unique arterial feeder, which was only borderline significant (*p* = 0.057) in the first analysis (birth-to-diagnosis), demonstrated statistical correlation with early hemorrhagic presentation (log-rank test, *p* = 0.037). Patients with AVMs with only one arterial feeder had a median time-to-ICH of 27 years, while those with more than one arterial feeder had an interval of 48 years. These findings correlate well with the R2ed AVM Score, published by Feghali et al., that included small size, exclusively deep venous drainage, and monoarterial feeding artery together with race and exclusively deep location as predicting factors of hemorrhagic presentation [21].

According to our analysis, small AVMs and those with exclusively deep venous drainage or unique arterial feeder, if diagnosed unruptured, should be considered for treatment, whenever possible, to prevent a future hemorrhage.

The ideal model to evaluate risk factors for hemorrhage is the one using the diagnosis-to-ICH timeline, following patients prospectively. The birth-to-diagnosis method needs several assumptions to be made, like survivorship bias and the congenital nature of the AVM [5]. The lesion is supposed to be present at birth, a theory that has been long debated, especially after several AVMs were diagnosed after birth, following different types of brain injury [28,29]. Karlsson et al. analyzed the age distribution of hemorrhage and found that AVMs in adults probably develop in the first years of life, posing a lower hemorrhage risk in the beginning. Furthermore, the authors state that AVM formation after the age of 25 is so rare that it has been published only in case reports [30]. This theory is in line with the observation made by Kim et al., who found that the birth-to-diagnosis curve and the diagnosis-to-ICH curve are most similar when shifting the birth-to-diagnosis timeline by 10 years. This might be due to several factors, like the dynamic character of the lesion and some hormonal changes that occur before puberty [5]. However, this pathology is very rare, and this method has the advantage of using a very long follow-up time and a larger number of ICH events, that cannot be gathered through the conventional method [5].The risk factors derived from the analysis using this method (both the birth-to-diagnosis and the 10 years-to-diagnosis timelines) are in line with those resulted from the univariate and multivariate analysis of hemorrhagic presentation. A time-dependent analysis might provide additional information regarding the probability of early hemorrhagic presentation.

Even if the birth-to-diagnosis method has its limitations, we consider it useful in gaining more insight into the natural history of brain AVMs, given the fact that long-term follow-up studies of conservatively treated lesions are scarce.

### 8.3. Epileptic Seizures in Brain AVMs

Seizures were the second most frequent presenting symptom after hemorrhage in stAVMs in our cohort. The seizure group had 77 patients, out of which 53 (68.8%) were unruptured AVMs. Understanding the pathogenesis of seizures in brain AVM patients is important in the management of all stAVMs but can be of great interest in patients diagnosed incidentally, or with minor symptoms, like headaches. Epileptic seizures represent an important burden regarding the quality of life, and the long-term safety of antiepileptic drugs in conservatively managed patients is yet to be evaluated, compared to interventional treatment. Unruptured AVMs, if untreated, pose not only a life-time risk of hemorrhage, but also the risk of a potentially debilitating symptom, like seizures. Josephson et al. estimated a 5-year risk of 58% of developing epilepsy after a first seizure in brain AVM patients [31].

In our cohort, seizures were independently associated with unruptured AVMs, exclusively superficial venous drainage and nidus size > 6 cm.

We did not find any statistical correlation between presenting seizures and the frontal lobe and sensorimotor areas [32], or temporal lobe [33]. However, similar to Shankar et al., we found that frontal, temporal and parietal locations together were highly associated with seizure occurrence in unruptured AVMs [34]. Although predilect locations vary between series, the connotation is similar: AVMs in contact with the neocortical parenchyma increase the risk of epileptic seizures. The correlation was significant both in univariate and multivariate analyses of the unruptured group, together with superficial venous drainage.

Garcin et al. found superficial venous drainage was associated with seizures, as well as male sex, large nidus, arterial border zone location, or the frontal lobe [35]. Although the mechanism is unclear, possible explanations, apart from the superficial location, could be the irritating effect of the draining vein on the surface of the cerebral cortex [35], or the cortical venous congestion produced by a long pial draining vein [34]. Ding et al. also analyzed the predictors of seizure presentation and found large nidus size, cortical location and unruptured lesions frequently associated with epileptic seizures [36].

Establishing risk factors for developing seizures, both in ruptured and unruptured supratentorial AVMs, from a large multi-center database might refine the indication for treatment, given the fact that microsurgical treatment has the highest chances of achieving seizure control [37], together with eliminating the hemorrhagic risk of these patients. Patients with brain AVMs at high-risk of developing seizures should be counseled not only about the risks and benefits of treatment over conservative management, but also regarding the burden of living with epilepsy.

## 9. Limitations

Our study has limitations. It is a single center, observational, retrospective study with inherent selection bias. The cohort is relatively small, and this fact might affect some of the statistical analysis’s accuracy. The birth-to-diagnosis time survival analysis, in comparison to the diagnosis-to-ICH method, cannot evaluate unruptured brain AVMs and their progression from diagnosis to a potential hemorrhage.

## 10. Conclusions

Hemorrhagic presentation and epileptic seizures are important manifestations of brain AVMs. Small AVMs, pediatric age and deeply located AVMs were statistically associated with the hemorrhagic presentation of brain AVMs. In birth-to-diagnosis survival analysis, small AVMs and those with exclusively deep venous drainage were independently associated with earlier hemorrhagic presentation. After shifting the birth-to-diagnosis curve by 10 years, unique arterial feeder demonstrated a positive correlation with earlier ICH presentation as well. Unruptured brain AVMs were statistically associated with seizures as a presenting symptom. Superficial venous drainage and frontal/temporal/parietal location significantly increase the risk of epileptic seizures as a presenting symptom in unruptured brain AVMs. When managing unruptured brain AVMs, it is important to consider the risk of developing seizures, in addition to the lifelong risk of hemorrhage, in determining the optimal treatment approach for each patient.

## Figures and Tables

**Table 1:** Baseline characteristic of patients with brain stAVMs in the study.

Characteristic	No. of Patients (%)
Females	83 (49.1)
Median age	36, range 5–79
Pediatric patients	27 (16)
Hemorrhagic presentation (at diagnosis)	78 (46.2)
Re-hemorrhage during follow-up period	4 (2.36)
First hemorrhage during follow-up period	1 (0.59)
Seizures	77 (45.6)
Early postoperative seizures	5 (3)
Seizures during follow-up	3 (1.77)
Motor deficits	40 (23.7)
Cranial nerve deficits	32 (18.9)
Headaches	141 (83.4)
Glasgow Coma Scale (GCS)	
≥14 pct	138 (81.6)
9–13 pct	21 (12.4)
≤8 pct	10 (6)

**Table 2:** Radiologic characteristics of stAVMs.

Characteristic	No. of Patients (%)
Supratentorial AVMs	169 (100)
AVM size	
<3 cm	91 (53.8)
3–6 cm	61 (36.1)
>6 cm	17 (10.1)
Nidus type	
Compact	151 (89.3)
Diffuse	18 (10.7)
Nidus localization	
Superficial	131 (77.5)
Deep	38 (22.5)
Venous drainage pattern	
Superficial	79 (46.7)
Deep (including mixt)	89 (52.7)
Undetermined	1 (0.6)
No. of drainage veins	
Unique	103 (61)
Multiple	45 (26.6)
Missing information	21 (12.4)
No. of arterial feeders	
1–2	80 (47.3)
>2	77 (45.6)
Missing information	12 (7.1)
Associated Aneurysms	23 (13.6)
SM grade	
SM I	22 (13)
SM II	50 (29.5)
SM III	57 (33.7)
SM IV	24 (14.2)
SM V	15 (9)
Undetermined	1 (0.6)

**Table 3:** Results of the Kaplan–Meier analysis comparing ICH-free probabilities in the birth-to-diagnosis time interval, considering different factors.

Group	No. of Cases	No. of AVMs Diagnosed with ICH	No. Censored (%)	Median ICH-Free Time in Years (95% CI)
Nidus size < 3 cm	91	51	40 (44)	41 (32–49)
Nidus size ≥ 3 cm	78	27	51 (65.4)	57 (43–70)
Cortical AVM	131	54	77 (58.5)	58 (44–72)
Deep AVM	38	24	14 (36.8)	40 (36–43)
Exclusively deep venous drainage	28	17	11 (39.3)	32 (22–42)
Superficial/mixt venous drainage	124	50	74 (59.7)	58 (46–70)
Multiple venous drainage	45	14	31 (68.9)	58
Unique venous drainage	103	50	53 (51.5)	46 (33–58)
Multiple arterial feeders	130	53	77 (59.2)	57 (47–66)
Unique feeding artery	27	16	11 (40.7)	36 (24–47)

**Table 4:** Results of the Kaplan–Meier analysis comparing ICH-free probabilities in the birth-to-diagnosis time interval, considering different factors, shifting the timeline with 10 years.

Group	No. of Cases	No. of AVMs Diagnosed with ICH	No. Censored (%)	Median ICH-Free Time in Years (95% CI)
Nidus size < 3 cm	85	46	39 (45.9)	31 (14, 47)
Nidus size ≥ 3 cm	75	25	50 (66.7)	47 (34, 60)
Superficial/mixt venous drainage	120	48	72 (60)	48 (36, 60)
Exclusively deep venous drainage	24	13	11 (45.8)	30 (18, 41)
Unique feeding artery	25	15	10 (40)	27 (6, 47)
Multiple arterial feeders	123	47	76 (61.8)	48 (37, 59)

## Data Availability

The data presented in this study are available on request from the corresponding author.
